# Marked skewing of entire T-cell memory compartment occurs only in a minority of CMV-infected individuals and is unrelated to the degree of memory subset skewing among CMV-specific T-cells

**DOI:** 10.3389/fimmu.2023.1258339

**Published:** 2023-10-26

**Authors:** Stephan Fuhrmann, Bernhard Reus, Oliver Frey, Alejandra Pera, Louis J. Picker, Florian Kern

**Affiliations:** ^1^Department for Hematopathology, Institute for Hematopathology Hamburg, Hamburg, Germany; ^2^Department of Informatics, School of Engineering and Informatics, University of Sussex, Brighton, United Kingdom; ^3^Institut für Laboratoriumsmedizin, Medizinische Hochschule Brandenburg, Brandenburg an der Havel, Germany; ^4^Immunology and Allergy Group, Maimonides Biomedical Research Institute of Cordoba (IMIBIC), University of Cordoba, Reina Sofia University Hospital, Cordoba, Spain; ^5^Department of Cell Biology, Physiology and Immunology, University of Cordoba, Cordoba, Spain; ^6^Vaccine and Gene Therapy Institute, Oregon Health & Science University, Beaverton, OR, United States; ^7^Department of Clinical and Experimental Medicine, Brighton and Sussex Medical School, Brighton, United Kingdom

**Keywords:** CMV, T cells, flow cytometry - methods, immunosenescence, memory T cell

## Abstract

**Background:**

Chronic CMV infection drives the clonal expansion and accumulation of terminally differentiated, dysfunctional CMV-specific T-cells. CMV infection also appears to accelerate the differentiation of non-CMV-specific T-cells; however, the extent of this phenomenon is unclear.

**Methods:**

The distribution of CD4 and CD8 T-cells into four memory subsets determined by CD45RA and CCR7 expression was analyzed in 96 CMV-infected (CMV+) and 81 CMV-uninfected (CMV-) older individuals. In CMV+ individuals, the distribution of IFN-γ producing CMV-specific T-cells into the same subsets was analyzed following stimulation with 16 different CMV antigens using flowcytometry (intracellular cytokine staining). We used previously published results to extrapolate the relative size of the entire CMV-specific CD4 and CD8 T-cell response from the summated response to selected antigens. The T-cell memory subset distribution across all CMV antigen-induced responses (weighted mean) was then used to calculate memory subset proportions (in % of CD4 or CD8 T-cells) of CMV-specific and non-CMV-specific T-cells. These were compared to the corresponding proportions in CMV- individuals.

**Results:**

Only a minority (20%–30%) of CMV+ individuals displayed overall proportions of terminally differentiated T-cell memory subsets above an upper outlier boundary defined in CMV- individuals. The calculated proportions of these subsets among non-CMV-specific T-cells in CMV+ individuals also exceeded the corresponding proportions in CMV- people, suggesting that their differentiation could be CMV-driven. In CMV+ people showing overall subset distributions within the outlier limits, the memory subset distributions of non-CMV-specific T-cells were more like those in CMV- people. Logistic regression revealed that CMV infection, age, and sex all had significant effects on one or more of the non-CMV-specific CD4 or CD8 T-cell memory subsets in CMV+ individuals, with CMV infection showing the strongest effect overall. Surprisingly, except for the CD45RA-/CCR7- CD4 T-cell subset, we only found weak correlations between corresponding memory subset proportions among all T-cells and CMV-specific T-cells.

**Conclusion:**

Our analysis supports an effect of CMV infection on non-CMV-specific T-cells; however, it is limited to a minority of individuals and not closely related to the degree of memory subset differentiation of CMV-specific T-cells. We propose that unknown predisposing factors might determine to what extent CMV infection affects non-CMV-specific T-cell differentiation.

## Introduction

The huge interest in Cytomegalovirus (CMV) as a driver of T-cell proliferation and differentiation originates from its apparent role in human immune system aging. During adult life, large numbers of CMV-specific T-cells are thought to undergo homeostatic proliferation and so maintain the overall size of the memory T-cell pool in the face of declining thymic output (1). In addition, frequent reactivation of CMV from latency provides repetitive antigen stimulation, thus further inflating the CMV-specific memory T-cell pool (2).

However, not only do CMV-specific memory T-cells increase with age in CMV-infected people, some authors have proposed that CMV infection also drives non-CMV-specific T-cells to differentiate towards a more terminal phenotype and so potentially modifies responses to other antigens in CMV-infected individuals (3). This was proposed because the difference in the numbers of advanced memory T-cells between CMV-infected and uninfected people appears to exceed what might reasonably be explained by the presence of CMV-specific T-cells alone.

To explore this phenomenon, Fletcher et al. examined CMV-specific T-cells as well as T-cells specific for *M. tuberculosis*, Epstein–Barr virus, varicella-zoster virus, and herpes simplex virus in both CMV- and CMV+ individuals (4). They showed that responses to these other infections were not only bigger among CMV+ individuals (in terms of CD4 T-cell percentage) but also more differentiated (larger proportions of CD27-/CD28- CD4 T-cells), suggesting that this might be direct evidence of an effect of CMV infection on non-CMV-specific T-cells. *In vitro* experiments suggested that such a bystander effect could be mediated by the production of IFN-α produced by plasmacytoid dendritic cells, and the authors speculated that this might occur *in vivo* in the T-cell areas of lymph nodes in the presence of CMV.

Given that >60% of the older population in Western Europe are CMV+ (with higher prevalence in most other parts of the world), understanding if CMV infection is responsible for the differentiation of non-CMV-specific T-cells is important because this might open up new therapeutic strategies focusing on CMV for counteracting inflammation. CMV-specific T-cells can be identified and enumerated using a number of different assays, with T-cell activation-based assays such as Elispot and flow cytometry being the most frequently used approaches (5, 6). These assays detect responses to different types of CMV antigen preparations such as CMV lysates and recombinant proteins, partial or complete proteins, and peptide pools. Irrespective of the type of assay, it is not possible, however, to measure the size of the immune response to the totality of CMV proteins in a single assay unless all expressed proteins are used at the same time. This is technically not feasible. As a result, in every CMV antigen-stimulated assay, there is always a population of CMV-specific T-cells of unknown size that was not activated by the antigens selected for stimulation. It is not possible to determine the relative size of that population. However, it is possible to run many assays covering multiple different CMV antigens in parallel in order to get close to the real proportion of CMV-specific T-cells in the blood. Using such an approach in 2005, we published the probably most comprehensive study ever done in terms of the CMV protein-specific T-cell response repertoire (7). Stimulations with >200 peptide pools covering all putative open reading frames (ORFs) of the CMV genome translating into amino acid (aa) chains >30 aa were run in parallel. The responses from all stimulations were added up to derive a summated, overall response to CMV in terms of CD4 and CD8 T-cell proportions. This produced the best possible (minimum) estimate of the entire CMV-specific T-cell response. One of the most important findings of this study was that this entire response can be approximated very closely using the sum of a reduced set of protein-specific responses. These included just six proteins for the CD4 and 15 proteins for the CD8 T-cell response, the sum of which showed correlations of *R* = 0.93 and *R* = 0.91, respectively, with the summated response to all proteins among CD4 and CD8 T-cells (7).

The present analysis was driven by the idea to capture the overall memory subset distributions of non-CMV-antigen-specific CD4 and CD8 T-cells in CMV+ people and compare these to the overall CD4 and CD8 T-cell distributions in CMV-negative people. This would show beyond doubt if there was a general effect of CMV infection on non-CMV-specific T-cells beyond responses to selected chronic infections. We attempt to do this by, first, estimating the size of the entire CD4 and CD8 T-cell responses to CMV (in % of all CD4 and all CD8 T-cells, respectively) based on the measured responses to a reduced set of CMV antigens as described above. In the second step, we determine the overall distributions of CD4 and CD8 T-cells in terms of naïve (*T*_Na_) central memory (*T*_CM_), effector memory (*T*_EM_), and revertant T-cells (*T*_Rv_) across all measured CMV antigen-specific responses. Third, by multiplying the estimated sizes of the entire CMV-specific CD4 and CD8 T-cell responses with each respective memory subset proportion, the contribution of CMV-specific T-cells to each of the CD4 and CD8 T-cell memory subsets (in terms of all CD4 or CD8 T-cells) is determined. Finally, by subtracting these estimated contributions from each of the memory subsets, the proportion of non-CMV-specific T-cells within each of them is revealed.

This analysis was carried out in 96 CMV-infected individuals, and the extrapolated memory subset distributions of the non-CMV-specific T-cells were compared to those in 81 CMV-uninfected individuals. We believe that our analysis provides an estimate of the memory subset distribution of non-CMV-specific T-cells in CMV-infected people that is as accurate as possible and corroborates the idea that CMV drives the differentiation of T-cells outside the CMV-specific compartment. At the same time, our analysis puts this effect into perspective and shows that strong effects are limited to a minority of CMV+ individuals.

## Materials and methods

### Methods

In this work, we reanalyzed data from a recent study, a part of which was previously published (“dataset 1”) (8). The study was approved by the UK National Research Ethics Service (NRES) “London Centre” (reference 13/LO/1270). Healthy CMV-positive (CMV+) and CMV-negative (CMV-) volunteers were recruited. Written informed consent was obtained from all participants.

Dataset 2 is from a previous study published in 2005 (7), which was approved by the Institutional Review Board of Oregon Health and Science University and included healthy CMV+ and CMV- adult volunteers, all of whom had provided written informed consent.

### Participants

Dataset 1 included 96 CMV+ and 81 CMV- people. The age distribution (years) was, for female patients, 69.8 ± 7.3 among CMV- and 71.0 ± 8.2 among CMV+ and, for male patients, 70.6 ± 7.6 (mean ± STD) among CMV- and 70.9 ± 6.6 among CMV+ individuals. CMV-specific responses were measured relative to 16 CMV antigens in terms of IL-2, TNF, and IFN-γ as well as basic memory phenotype data (CD45RA and CCR7 expression) as described below. We also used the results from 33 CMV+ individuals in dataset 2 (intracellular IFN-γ production following stimulation with CMV antigens). The age distribution in dataset 2 (years) was, for female patients, 39.1 ± 8.9 and, for male patients, 34.0 ± 9.1 (mean ± STD).

### Activation assays and flow cytometry

Briefly, for dataset 1, peripheral blood mononuclear cells were isolated from peripheral blood using Ficoll–Hypaque density gradient centrifugation and stimulated overnight with 14 overlapping peptide pools representing 16 CMV proteins (**Table 1**). The cells were stained with phenotype markers (CD3, CD4, CD8, CD45RA, and CCR7) and intracellular activation markers, including IL-2, TNF, and INF-γ. Samples were acquired by flow cytometry, and data were processed using FlowJo (v9.6).

**Table 1:** Correlation of selected CMV protein activation with total response.

	Selection in original study	Correlation with total response^a^	Selection in recent study	Correlation with total response
CD4	UL55, UL83, UL86, UL99, UL153 (Towne), UL32	0.922 (0.42)	UL55, UL83, UL86, UL153 (Towne), UL32, UL36, US24	0.925 (0.35)
CD8	UL123, UL83, UL122, UL28, UL48, US3, UL151 (Toledo), UL82, UL94, US29, UL99, UL103, US32, US24, UL36	0.907 (0.67)	UL123, UL83, UL122, UL28, UL48, US3, UL151 (Toledo), UL82, UL94, US29, US24, UL36, UL32, UL55, UL153 (Towne), UL86	0.887 (0.58)

The underlined proteins differ between cohorts.

a Total response refers to the response to 203 CMV ORFs in the original study.

**Supplementary Figure S1** illustrates the gating strategy used. The percentages of activated T-cells determined in stimulated tubes were corrected by subtracting the corresponding percentages in unstimulated tubes. The percentages provided for activated CD4 T-cells and their subsets always refer to all CD4 T-cells; the same applies to CD8 T-cells. The memory compartments were defined based on the expression of CD45RA and CCR7. Four differentiation stages were defined as follows: naïve (*T*_Na_, CD45RA+CCR7+), central memory (*T*_CM_, CD45RA-CCR7+), effector memory (*T*_EM_, CD45RA-CCR7-), and revertant (*T*_Rv_, CD45RA+CCR7-). For dataset 2, the methods were described in detail in Sylwester et al. in 2005 (7). A very similar protocol was followed, albeit with intracellular staining for IFN-γ only.

### Calculations

#### CMV-reactive and non-CMV-reactive T-cells

The calculation of CMV-specific and non-CMV-specific complementary subsets was performed as follows. *A* is the frequency of all CD4 T-cells, *B* is the frequency of all CD8 T-cells, and for the purpose of this study, where all proportions are expressed in terms of *A* and *B*, both are set to 1 (100%), with all subpopulations being expressed as fractions of 1 (i.e., 0.45 = 45%).

The frequency of the subpopulation of CD4 or CD8 T-cells, respectively, responding with activation to a CMV-antigen *i* (also CMV_i_) is: ACMV*_i_* and BCMV*_i_*

The frequency of all CD4 or CD8 T-cells not responding to a CMV-antigen *i* in a given experiment is defined as follows: ACMV*_i_^C^* and BCMV*_i_^C^* for CD4 and CD8 T-cells, respectively.

For each experiment using a CMV-antigen *CMV_i_*, the sum of CD4 T-cells is the sum of cells responding or, alternatively, not responding to one of the *n*=16 CMV antigens used in the study, i.e., it is:


$$A=\sum_{i=1}^{16}ACMV_{i}+ACMV_{i}^{C}$$

While individuals may respond to additional CMV antigens not tested here, for the purpose of this study, we assume that:


$${\text{ACMV}}_{\text{all}}=\sum_{i=1}^{16}{\text{ACMV}}_{i}$$

is the total CD4 T-cell response to CMV. In analogy:


$${\text{BCMV}}_{\text{all}}=\sum_{i=1}^{16}{\text{BCMV}}_{i}$$

is the total CD8 T-cell response to CMV.

The proportion of non-CMV-reactive CD4 or CD8 T-cells is the number of all T-cells minus the number of all reactive T-cells:


$${\text{ACMV}}_{\text{all}}^{\text{C}}=\text{A}-\sum_{i=1}^{16}{\text{ACMV}}_{i}$$

and in analogy


$${\text{BCMV}}_{\text{all}}^{\text{C}}=\text{B}-\sum_{i=1}^{16}{\text{BCMV}}_{i}$$

The frequency of cells not responding to the CMV antigens used for stimulation can only be calculated as the overall frequency of CD4 or CD8 T-cells (CD4 T_all_ and CD8 T_all_) minus the sum of the frequencies of CMV-specific CD4 or CD8 T-cells responding to the 16 CMV-antigens used, respectively.

**Figure 1:**
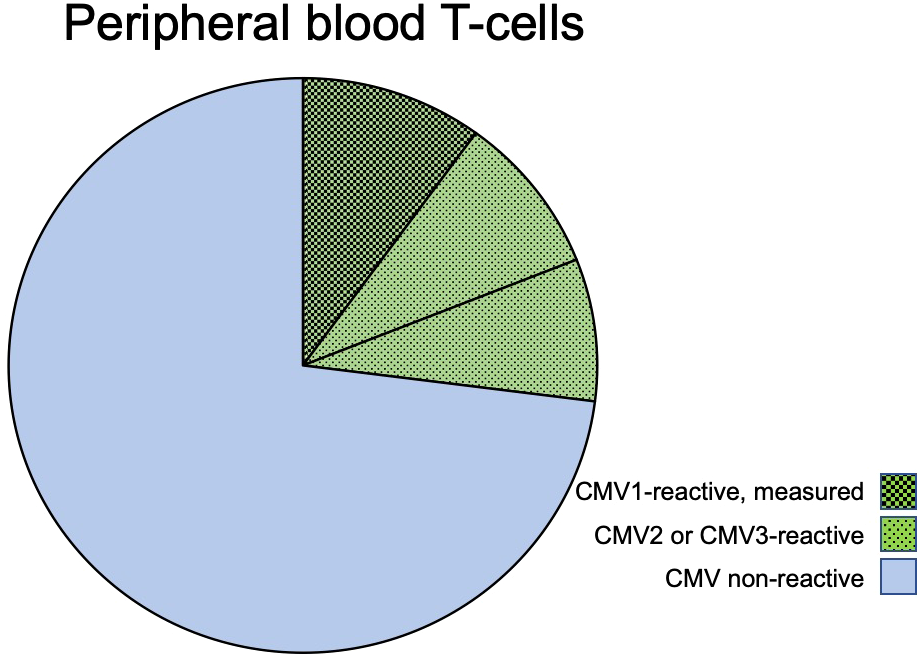
CMV-reactive and CMV non-reactive T-cells. The pie chart shows the composition of T-cells (CD4 or CD8) in regard to CMV-reactive and CMV non-reactive cells in a *hypothetical* individual who has T-cell responses to three given CMV proteins: CMV1, CMV2, and CMV3. The block of green-shaded pie slices represents CMV-reactive T-cells that will respond with activation when exposed to these CMV antigens. The blue, non-shaded slices represent T-cells that will not respond to these or other CMV antigens. When stimulated with CMV1, only cells responding to this antigen will be activated (slices with darkest shading); however, the cells that would be responding to antigens CMV2 and CMV3 (lighter shading) are non-responsive when stimulated with CMV1. In an experiment using only CMV1 for stimulation, the T-cells reactive to CMV2 and CMV3 will, therefore, automatically be counted into the non-CMV-reactive T-cells.


Note that the sum of all CMV-non-reactive T-cells in an individual and with respect to the tested antigens is *not* the sum of all non-reactive T-cells from experiments 1–16 because, in each experiment, the response to only one antigen is tested and cells not responding to that antigen may still respond to another antigen when stimulated with it (**Figure 1**).

$$\text{ACM}V_{all}^{C}=\text{A}-\sum_{i=1}^{16}\text{ACM}{\text{V}}_{\text{i}}\neq \sum_{i=1}^{16}\text{ACM}{\text{V}}_{\text{i}}^{\text{C}}$$

In analogy, for CD8 T-cells


$$\text{BCM}V_{all}^{C}=\text{B}-\sum_{i=1}^{16}\text{BCM}{\text{V}}_{\text{i}}\neq \sum_{i=1}^{16}\text{BCM}{\text{V}}_{\text{i}}^{\text{C}}$$

### Phenotypic distribution

**Figure 2:**
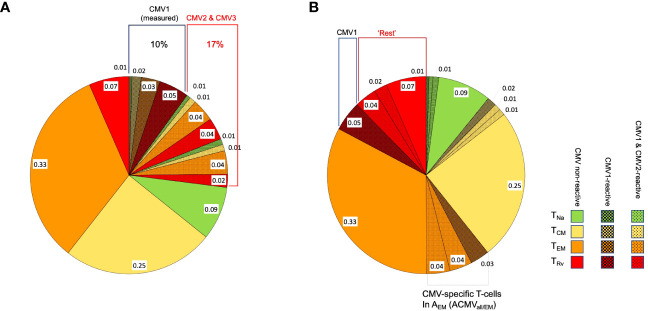
Distribution of phenotypic T-cell subsets. The pie charts show the division of CD4 or CD8 T-cells with respect to the phenotypical subsets, T_Na_ (naïve, green), T_CM_ (central memory, yellow), T_EM_ (effector memory, orange), and T_Rv_ (revertant, red), in a hypothetical case for illustration. **(A)** The block of shaded pie slices represents CMV-reactive T-cells, with dark-shaded slices representing CMV1-reactive T-cells and light-shaded slices representing CMV2 and CMV3-reactive T-cells. The non-shaded slices represent T-cells that will not respond to any of these antigens. When stimulation with CMV1 occurs, only cells responding to this antigen will be activated, but CMV2 and CMV3-reactive T-cells will be indistinguishable from non-CMV-reactive T-cells and contribute to their apparent memory subset distribution. This is further illustrated under **(B)**. **(B)** The slices shown under **(A)** are sorted by T-cell memory subset. When the proportion of CMV1-reactive T_Rv_ cells is subtracted from the overall proportion of T_Rv_ cells (proportions with respect to all CD4 or all CD8 T-cells), the remaining T_Rv_ cells are composed of CMV non-reactive and also CMV1- and CMV2-reactive T-cells. In order to reveal the true proportion of CMV-non-reactive T-cells in each T-cell memory compartment (non-shaded areas of the pie), the proportions of *all* CMV-reactive T-cells in each of these compartments must be subtracted from its respective overall size.

The overall distribution of the phenotypic subsets referred to as *T*_Na_, *T*_CM_, *T*_EM_, and *T*_Rv_, among *A* and *B* results from the composition of these subsets among ${\text{ACMV}}_{\text{all}}$ and ${\text{ACMV}}_{\text{all}}^{\text{C}}$ (**Figures 2A, B**). In the interest of space, we continue the explanations for CD4 T-cells (*A*) only since they are exactly analogous for CD8 T-cells (*B*).

Because *n* = 16 antigens were used and the size of each phenotypic subset is given as a fraction of all CD4 T-cells, the following applies, where ${\text{ACMV}}_{i/\text{Na}}$ , ${\text{ACMV}}_{i/\text{CM}}$ , ${\text{ACMV}}_{i/\text{EM}}$ , and ${\text{ACMV}}_{i/\text{Rv}}$ denote the sizes of the phenotype subsets among $ACMV_{i}$ , respectively.


$${\text{ACMV}}_{\text{all}/\text{Na}}=\sum_{i=1}^{16}{\text{ACMV}}_{i/\text{Na}}$$

is the total response to CMV in the naive compartment.

The sum of all CMV-reactive cells is the sum of sub-sums with respect to each phenotypic subset.


$${\text{ACMV}}_{\text{all}}=\sum_{i=1}^{16}{\text{ACMV}}_{i/\text{Na}}+\sum_{i=1}^{16}{\text{ACMV}}_{i/\text{CM}}+\sum_{i=1}^{16}{\text{ACMV}}_{i/\text{EM}}+\sum_{i=1}^{16}{\text{ACMV}}_{i/\text{Rv}}$$

Given that in each experiment cells not responding to the CMV-antigen used may still respond to another CMV antigen, the size of ${\text{CMV}}_{\text{all}/\text{Na}}^{\text{C}},\text{ f}$ or example, cannot be determined as the sum of naïve T-cells among the non-responding cells in each experiment (**Figures 2A, B**).

In analogy to the determination of all non-CMV-reactive T-cells mentioned above, the proportion of each phenotype subset, *T*_Na_, *T*_CM_, *T*_EM_, and *T*_Rv_, among non-CMV-reactive T-cells ( ${\text{ACMV}}_{\text{Na}}^{\text{C}}$ , ${\text{ACMV}}_{\text{CM}}^{\text{C}},{\text{ ACMV}}_{\text{EM}}^{\text{C}},{\text{ and ACMV}}_{\text{Rv}}^{\text{C}}$ ) is determined by subtracting from the overall proportion of the relevant subset (e.g., *A*_Na_) the summated proportions of this phenotypic subset among CMV-specific T-cells across all stimulations.


$${\text{ACMV}}_{\text{Na}}^{\text{C}}=A_{\text{Na}}-\sum_{i=1}^{16}{\text{ACMV}}_{i/\text{Na}}$$

$${\text{ACMV}}_{\text{CM}}^{\text{C}}=A_{\text{CM}}-\sum_{i=1}^{16}{\text{ACMV}}_{i/\text{CM}}$$

$${\text{ACMV}}_{\text{EM}}^{\text{C}}=A_{\text{EM}}-\sum_{i=1}^{16}{\text{ACMV}}_{i/\text{EM}}$$

$${\text{ACMV}}_{\text{Rv}}^{\text{C}}=A_{\text{Rv}}-\sum_{i=1}^{16}{\text{ACMV}}_{i/\text{Rv}}$$

The overall percentage of non-CMV-reactive CD4 T-cells is the sum of the non-CMV-reactive T-cells in the four phenotypic compartments.


$${\text{ACMV}}^{\text{C}}={\text{ACMV}}_{\text{Na}}^{\text{C}}+{\text{ACMV}}_{\text{CM}}^{\text{C}}+{\text{ACMV}}_{\text{EM}}^{\text{C}}+{\text{ACMC}}_{\text{Rv}}^{\text{C}}$$

By dividing the proportion of non-CMV-reactive CD4 T-cells in each compartment by ${\text{ACMV}}_{\text{all}}^{\text{C}}$ , the proportion of each phenotypic subset is normalized so that the sum is 1. This provides the distribution of the phenotypic subsets among non CMV-reactive T-cells.


$${\text{ACMV}}_{\text{Na}/\text{norm}}^{\text{C}}={\text{ACMV}}_{\text{Na}}^{\text{C}}/{\text{ACMV}}^{\text{C}}$$

$${\text{ACMV}}_{\text{CM}/\text{norm}}^{\text{C}}={\text{ACMV}}_{\text{CM}}^{\text{C}}/{\text{ACMV}}^{\text{C}}$$

$${\text{ACMV}}_{\text{EM}/\text{norm}}^{\text{C}}={\text{ACMV}}_{\text{EM}}^{\text{C}}/{\text{ACMV}}^{\text{C}}$$

$${\text{ACMV}}_{\text{Rv}/\text{norm}}^{\text{C}}={\text{ACMV}}_{\text{Rv}}^{\text{C}}/{\text{ACMV}}^{\text{C}}$$

$${\text{ACMV}}_{\text{Na}/\text{norm}}^{\text{C}}+{\text{ACMV}}_{\text{CM}/\text{norm}}^{\text{C}}+{\text{ACMV}}_{\text{EM}/\text{norm}}^{\text{C}}+{\text{ACMV}}_{\text{Rv}/\text{norm}}^{\text{C}}=1$$

### Statistical analysis

Statistical analysis was performed using the SPSS 27 software package (IBM). For comparisons of subset sizes, a non-parametric test for independent samples (Mann–Whitney) was used unless otherwise indicated. Linear regression was performed using the general linear model.

## Results

We used two datasets for this analysis: first, our most recent dataset (“dataset 1”), which included 96 CMV+ and 81 CMV- people with CMV-specific responses measured relative to 16 CMV antigens in terms of IL-2, TNF, and IFN-γ as well as basic memory phenotype data (CD45RA and CCR7 expression), and second, we used the original dataset from 2005 (“dataset 2”), including T-cell responses to 213 putative CMV proteins (CMV ORFs translated into protein sequences, represented by 13,687 peptides arranged in pools) in terms of IFN-γ. This dataset included 16 female and 17 male CMV+ participants of mixed biogeographical ancestry (for details, see “Materials and methods”).

**Figure 3:**
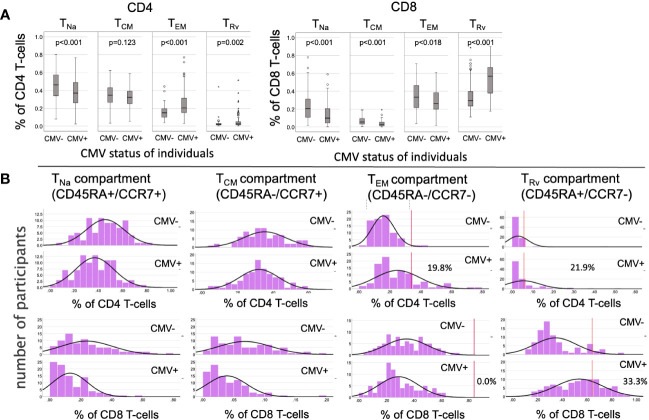
Frequencies and distributions of memory T cell subsets. **(A)** Frequencies of CD4 (left) and CD8 (right) memory T-cell subsets. **(B)** Distribution of T-cell memory subsets, with one column for each subset. The upper panel of each diagram shows CD4, while the lower panel shows CD8 T-cells and their distribution among the CMV- (rows 1 and 3) and CMV+ (rows 2 and 4) populations. The red lines in columns 3 and 4 (counting from the right) indicate the upper outlier boundary defined among CMV- people and calculated as upper quartile + 1.5 * interquartile range (UQ + 1.5 * IQR).

We initially analyzed the distribution of canonical memory T-cell subsets (9) in dataset 1 to assess the differences between CMV+ and CMV- people in detail. **Figure 3A** shows a comparison between the percentages of naïve T-cells (CD45RA+/CCR7+, *T*_Na_), central memory T-cells (CD45RA-/CCR7+, *T*_CM_), effector memory T-cells (CD45RA-/CCR7-, *T*_EM_), and CD45RA-revertant memory T-cells (CD45RA+/CCR7-, *T*_Rv_) among the CD4 (**left**) and CD8 (**right**) T-cell compartments in CMV- and CMV+ people. Differences between CMV- and CMV+ individuals were statistically significant with respect to all of these subsets and in both T-cell compartments, except for CD4 T_CM_ cells. The biggest differences among CD4 T-cells were found with respect to the T_EM_ compartment, whereas among CD8 T-cells the biggest differences were seen in the T_Rv_ compartment. We also visualized the overall distributions of T-cell frequencies across these compartments using histograms (**Figure 3B**). In keeping with our previous work (10), we used CMV- individuals to determine the upper boundaries for outliers and applied them to both CMV- and CMV+ individuals as this approach provides a sense of how often CMV+ individuals show distributions that would not be expected among CMV- individuals. Upper outlier boundaries were calculated as upper quartile + 1.5 * interquartile range (UQ + 1.5 * IQR). For the most part, the T-cell frequencies measured in each compartment in CMV+ donors were inside the outlier boundaries defined in CMV- individuals (indicated as vertical red lines for *T*_EM_ and *T*_Rv_ cells) (**Figure 3B**). Among CD4 T-cells, the *T*_EM_ and *T*_Rv_ compartments showed the most striking differences between CMV+ and CMV- people. The distribution of *T*_EM_ and *T*_Rv_ CD4 T-cells among CMV+ individuals compared with CMV- people appeared more skewed (i.e., increase of the width of the distribution and loss of basic curve shape) than shifted (i.e., change of mean while retaining the basic shape of the curve) and showed many outliers (**Figure 3B**). CD8 T_Rv_ cells also showed strong population skewing with a majority of CMV+ individuals, however, not showing outlier values (**Figure 3B**). This initial analysis demonstrated that an effect of CMV on the T-cell memory phenotype distribution does not occur in all CMV+ people to the same extent. A majority of individuals do not show such a marked effect at all (**Table 2**). Of note is that, in approximately 2/3 of CMV+ people, the proportions of T-cells remained within the respective outlier limits found in CMV- people in all analyzed compartments (64% in CD4 and 65% among CD8).

**Table 2:** Peripheral blood T-cell memory subset distribution: outliers among CMV- and CMV+ people.

T-cell subset	Compartment	CMV- (n = 81)		CMV+ (n = 96)	
		Outlier count	Outlier^a^	Outlier count	Outlier
CD4	Na	0	0.0%	0	0.0%
CD4	CM	0	0.0%	0	0.0%
CD4	EM	2	2.5%	19	**19.8%**
CD4	Rv	4	4.9%	21	**21.9%**
CD8	Na	2	2.5%	0	0.0%
CD8	CM	1	1.2%	1	1.0%
CD8	EM	0	0.0%	0	0.0%
CD8	Rv	7	8.6%	32	**33.3%**

a Outliers (non-parametric) were defined among CMV- individuals as upper quartile + 1.5 * interquartile range.High percentages of outliers of subset compartments above 15% are boldface.

### Variable contributions of CMV-specific T-cells to the canonical CD4 and CD8 memory T-cell subsets

**Figure 4:**
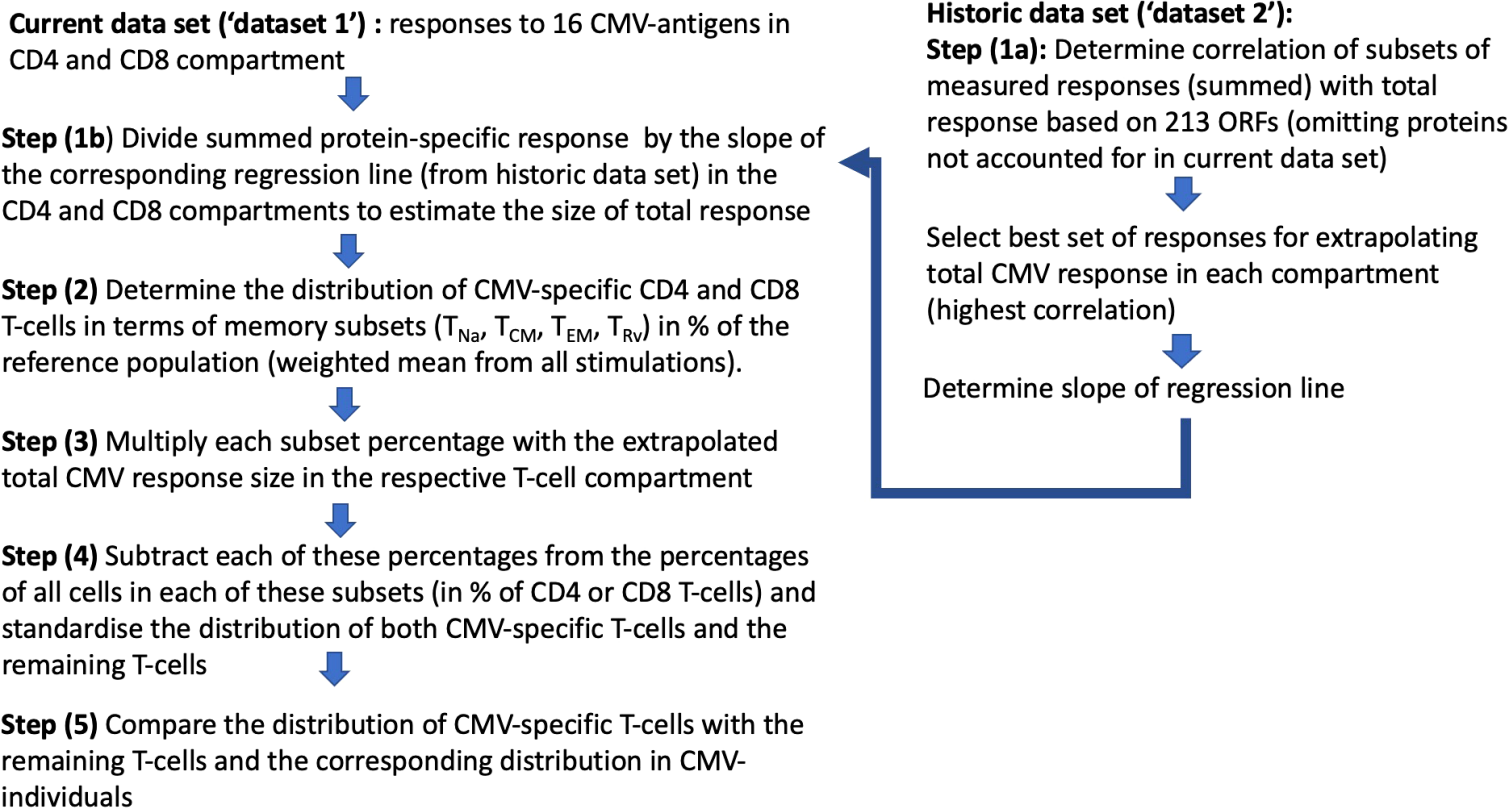
Step-by-step analysis workflow: Two datasets were used, with “dataset 1” being the current and “dataset 2” as the historical dataset. Dataset 2 was used to determine the factor by which the size of the T-cell response measured with respect to proteins in dataset 1 had to be multiplied in order to estimate the overall CMV-specific T-cell responses (1/slope of the regression line; see **Figure 5**).

As a next step, we wanted to obtain a realistic measure of the size of the actual, direct contribution of CMV-specific T-cells to these compartments. This required, first, estimating the size of the entire CMV-specific CD4 and CD8 T-cell response and, second, its memory T-cell subset distribution. For this analysis, we needed both datasets. Whereas dataset 1 provided the values to be extrapolated, dataset 2 was required to determine the parameters used in the extrapolation (**Figure 4**). With respect to dataset 2, the CMV proteins used for stimulation were originally ranked by (i) the summated CD4 T-cell response and (ii) the summated CD8 T-cell response in all participants. Beginning with the protein in rank 1 and adding proteins one by one in the order of the ranking, the smallest number of protein-specific responses whose sum correlated with an *R* > 0.9 with the overall CD4 or CD8 T-cell response, respectively, was determined. Responses to the top six CD4 T-cell target proteins provided a correlation coefficient of *R* = 0.922, and responses to the top 15 CD8 proteins provided *R* = 0.908. With respect to CD4 T-cells, the response relative to these six proteins corresponded to 0.42 × the response to all proteins (slope of regression line), whereas the response to the top 15 CD8 T-cell targets amounted to 0.67 × the response to all proteins (7). We were unable, however, to adopt these originally calculated values exactly because dataset 1 did not include responses to three of the CMV proteins included in the top six CD4 and/or top 15 CD8 antigens arising from the original analysis (underlined proteins in **Table 1**). They had been removed from our UK stimulation panel because they had never stimulated a robust response in our previous UK cohorts. Our analysis, therefore, included additional steps requiring us to revisit dataset 2 in order to repeat the original analysis based on the proteins included in our UK panel. The entire analysis was thus carried out in five steps (**Figure 4**) as discussed below.

**Figure 5:**
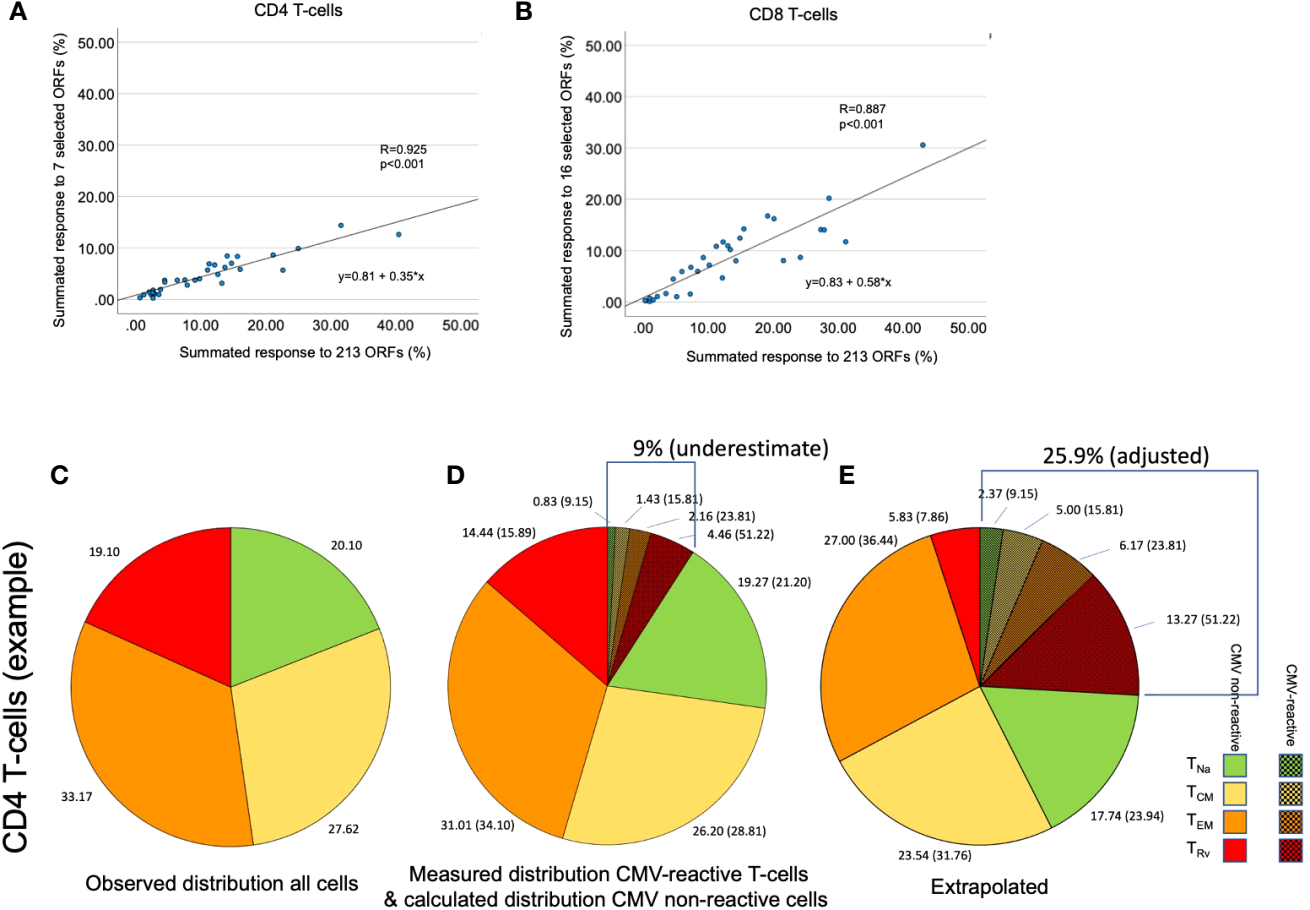
Determination of the size of the overall CMV-specific T-cell response and its memory subset distribution. **(A, B)** The size of the summed CMV response to 213 tested ORFs was plotted against the response to seven [for CD4, **(A)**] or 16 [for CD8, **(B)**] immunodominant proteins in dataset 2. This was done to reproduce the originally published analysis with the present set of proteins. **(C)** In an example shown for illustration purposes, the distribution of the four CD4 memory T-cell subsets, T_Na_, T_CM_, T_EM_, and T_Rv_, was determined by the combination of CD45RA und CCR7 staining. **(D)** CMV-specific T-cell responses were quantified by stimulation with a set of CMV peptide pools representing the seven selected CD4 target proteins. Intracellular IFN-γ staining in combination with phenotype staining under **(C)** permitted the determination of the four memory subsets with respect to each stimulation. The shaded slices show the summated responses across all stimulations. However, this sum does not represent the size of the entire CMV-specific response. **(E)** The extrapolation of the overall response (9.01/0.35 = 25.90) and the proportion of each memory subset in that response is illustrated. The memory subset distribution of CMV-specific T-cells affects the calculated distribution of all remaining non-CMV-specific T-cells.

In step (1), we determined how well the protein-specific responses (or subsets of these) tested for dataset 1 would predict the overall response to CMV according to the historical dataset 2, which comprised the entire response. In step (1a), the selection of proteins was carried out in the same way as done originally, however, leaving out proteins not tested for dataset 1. We found that the peptide pools covering UL55, pp65, UL86, UL32, and UL36/US24 achieved the best correlation of *R* = 0.925 with the overall CD4 T-cell response (*p* = 0.001, slope 0.35), whereas all studied proteins achieved a correlation of *R* = 0.889 with the overall CD8 T-cell response (*p* = 0.001, slope 0.59) (**Table 1**; **Figures 5A, B**). In step (1b), we divided the summed CD4 T-cell responses to UL55, pp65, UL86, UL32, and UL36/US24 by the slope of the regression line, i.e., 0.35, and in analogy the summed CD8 T-cell responses (all pools) by 0.58. This provided the best possible estimate of the overall proportion of CD4 and CD8 T-cells responding to CMV with respect to dataset 1.

In step (2), we determined the overall memory subset distribution of CMV-specific T-cells for each CMV+ individual by adding up the percentages of IFN-γ-producing T-cells (in terms of CD4 or CD8 T-cells) obtained with each protein stimulation and separately for *T*_Na_, *T*_CM_, *T*_EM_, and *T*_Rv_. The sum of these memory-compartment-specific responses across all stimulations equaled the overall proportion of CD4 and CD8 responding to these proteins in any given individual. For both CD4 and CD8 T-cells, the summed percentages of each memory subset across all CMV protein-specific responses is the overall proportion contributed by each memory subset to the entire response. This “summative” approach gives more weight to larger responses and less weight to smaller responses (weighted average) (**Supplementary Table S1**).

In step (3), we determined the probable memory subset distribution of the total CMV-specific response. To this end, the estimated overall percentage of CD4 and CD8 T-cells determined in step (1) was multiplied with the percentages (weighted average) of CD4 and CD8 T-cells in each memory subset as determined in step (2). **Figures 5C–E** illustrate that these calculations affect the estimated size of both CMV-specific and non-CMV-specific CD4 and CD8 T-cell memory subsets.

**Figure 6:**
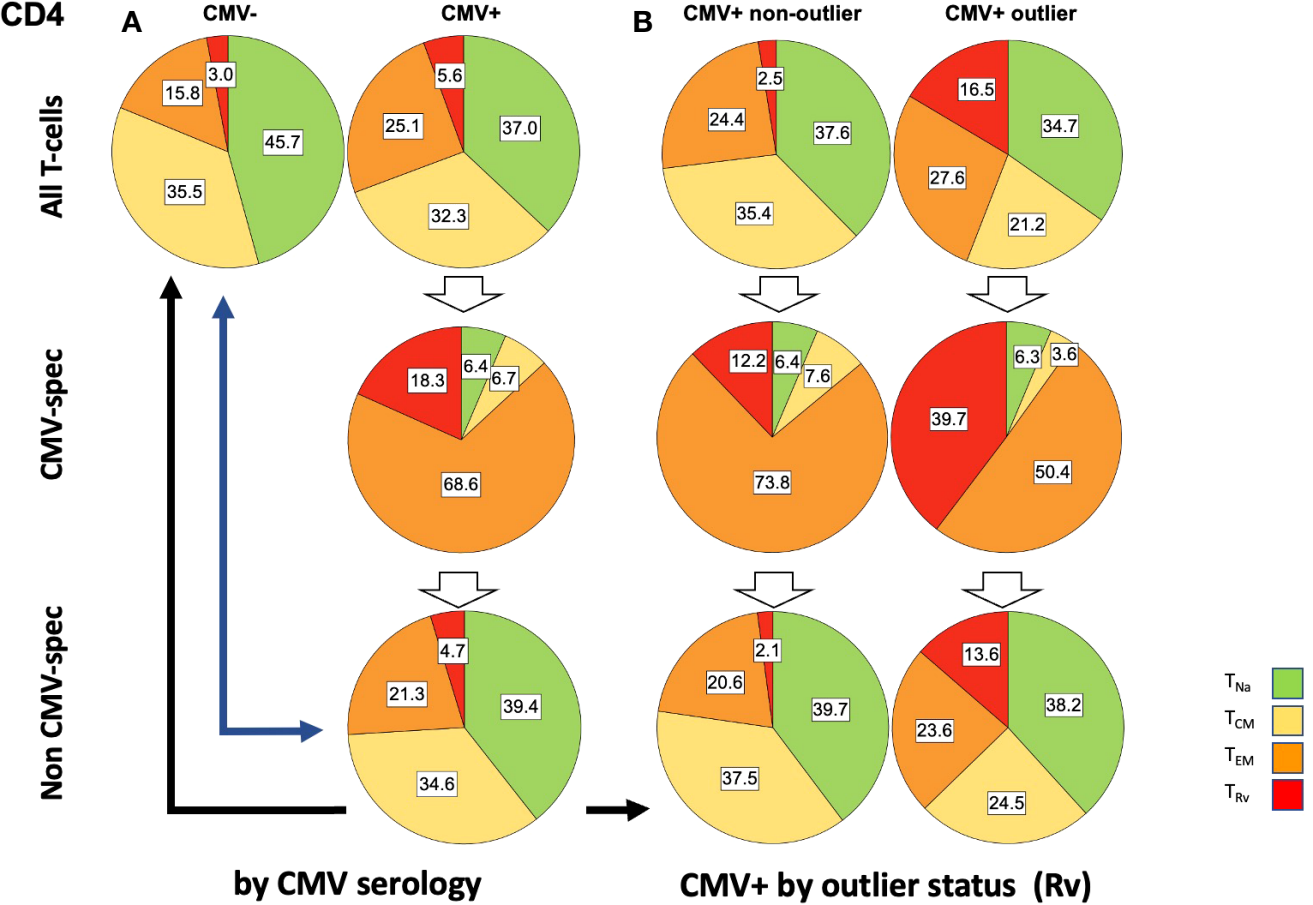
Relative T-cell memory compartment distributions among all CD4 T-cells, CMV-specific CD4 T-cells, and non-CMV-specific CD4 T-cells. The pie charts show the T-cell memory compartment distributions across all CD4 T-cells (top), CMV-specific CD4 T-cells (middle), and non-CMV-specific CD4 T-cells (bottom, calculated/extrapolated). **(A)** Comparison according to CMV status. **(B)** Comparison according to outlier status (the outlier status refers to the size of CD4 T_Rv_ compartment according to **Figure 3B**; right). The arrows indicate relevant comparisons between extrapolated non-CMV-specific T-cell memory compartments in CMV+ people and those in CMV- people.

**Figure 7:**
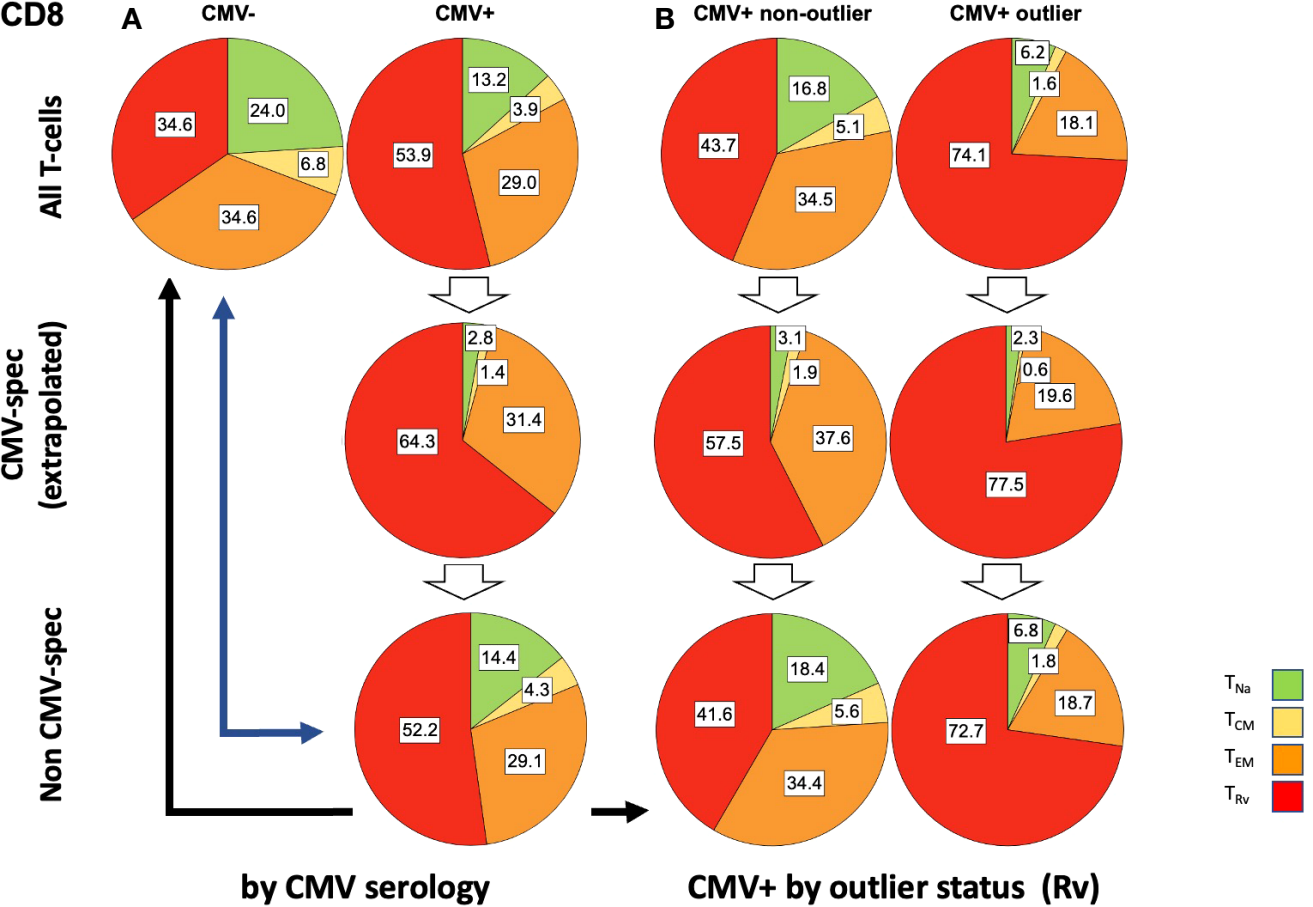
Relative T-cell memory compartment distributions among all CD8 T-cells, CMV-specific CD8 T-cells, and non-CMV-specific CD8 T-cells. The pie charts show the T-cell memory compartment distributions across all CD8 T-cells (top), CMV-specific CD8 T-cells (middle), and non-CMV-specific CD8 T-cells (bottom, calculated/extrapolated). **(A)** Comparison according to CMV status. **(B)** Comparison according to outlier status (the outlier status refers to the size of CD8 T_Rv_ compartment according to **Figure 3B**; right). The arrows indicate relevant comparisons between extrapolated non-CMV-specific T-cell memory compartments in CMV+ people and those in CMV- people.

In step (4), the extrapolated CMV-specific portion of each memory subset was subtracted from the measured percentage of each subset (in an unstimulated tube), leaving the estimated percentage of non-CMV-specific T-cells with respect to each memory subset in terms of CD4 or CD8 T-cells—for example: all CD4 T_EM_ cells (in % of CD4) - CMV-specific T_EM_ cells (in % of CD4) = non-CMV-specific T_EM_ cells (in % of CD4). In order to derive separate memory subset distributions for CMV-specific and non-CMV-specific T-cell populations from the estimated percentages, the percentages were standardized within each population (i.e., their sum was set to 100%, and the percent contribution of each subset to the total was calculated) (**Figures 6A, B**, **7A, B**).

In step (5), we compared the memory subset distributions of the estimated non-CMV-specific T-cell populations in CMV+ individuals with the corresponding distributions in CMV- individuals (**Figures 6**, **7**). Compared with the overall CD4 memory T-cell subset distribution in CMV+ individuals, the calculated distribution of non-CMV-specific CD4 T-cells was more similar to the overall CD4 memory T-cell subset distribution among CMV- people (**Figure 6A**, top left vs. top right, and top left *versus* bottom right, blue arrow). A similar result was observed when an outlier status was assigned according to the size of the CD4 T_EM_ compartment (**Supplementary Figure S2**). With respect to CD8 T-cells, there was less similarity between the respective distributions (**Figure 7A**, top left *versus* bottom right, blue arrow). Differences [non-CMV-specific T-cells in CMV+ people *versus* overall memory subsets in CMV- people] were statistically significant for the CD4 *T*_Na_ (*p* = 0.008) and *T*_EM_ (*p* = 0.001) subsets as well as the CD8 *T*_Na_ (*p*< 0.001), *T*_CM_ (*p*< 0.001), *T*_EM_ (*p* = 0.022), and *T*_Rv_ (*p*< 0.001) subsets.

Because the main effects of CMV on the overall memory subset distribution occurred in a group of individuals with outlier values in terms of *T*_EM_ and *T*_Rv_ in CD4 T-cells and *T*_Rv_ in CD8 T-cells, we also compared the estimated distributions of non-CMV-specific T-cells in those with and without outliers to the overall distributions among CMV- people (**Figures 6B**, **7B**). The calculated memory subset distributions of non-CMV-specific T-cells in CMV+ individuals without *T*_Rv_ outlier values among CD4 (**Figure 6A**, top left, *versus* **Figure 6B**, bottom left, black arrow) or CD8 T-cells (**Figure 7A**, top left, *versus* **Figure 7B**, bottom left, black arrow) showed greater similarity to the distributions among CMV- people compared to when all CMV+ individuals were included. The same effect was also observed when an outlier status was assigned according to the size of the CD4 *T*_EM_ compartment (**Supplementary Figure S2**).

We further used linear regression (general linear model) to estimate the effect of CMV status, age, and gender on the extrapolated size of each of the corrected non-CMV-specific *T*_CM_, *T*_EM_, and *T*_Rv_ subsets (**Tables 3**, **4**). This was done to investigate if the size of these compartments would still be affected by CMV serology, indicating an effect of CMV on (supposedly) non-CMV-specific populations. For CD4, the effect of positive CMV serology on the *T*_EM_ subset was statistically significant, but not its effect on the *T*_CM_ or *T*_Rv_ subsets. Being CMV+ increased the *T*_EM_ subset by approximately 5.6% on average (*p*< 0.001). A significant effect of gender was observed on the CD4 *T*_EM_ subset, indicating that, in women, this subset was approximately 4.6% smaller than in men (*p* = 0.002) (**Table 3**).

**Table 3:** Effect of CMV, age, and sex on CD4-”corrected” populations.

Sample	Beta coefficient (95% CI)	Std. error	*p*-value
	Model: CMV serology, age, sex		
CMV serology			
T_Na_	-.063 (-.109, -.017)	.0235	**.008**
T_CM_	-.009 (-.045,.026)	.0181	.617
T_EM_	.056 (.027,.085)	.0149	**<0.001**
T_RV_	.016 (-.004,.035)	.0101	.118
Age			
T_Na_	-.001 (-.004,.002)	.0016	.465
T_CM_	.000 (-.003,.002)	.0012	.734
T_EM_	.001 (-.001,.003)	.010	.306
T_RV_	.001 (-.033,.043)	.0007	.422
Sex (female)			
T_Na_	.069 (.023,.115)	.0234	**.003**
T_CM_	-.005 (-.041,.030)	.0181	.765
T_EM_	-.046 (-.075, -.017)	.0148	**0.002**
T_RV_	-.017 (-.037,.003)	.019	.087

P-values in boldface are below the 0.05 threshold.

**Table 4:** Effect of CMV, age, and sex on CD8-”corrected” populations.

Sample	Beta coefficient (95% CI)	Std. error	*p*-value
	Model: CMV serology, age, sex		
CMV serology			
T_Na_	-.092 (-.134, -.051)	.0212	**<0.001**
T_CM_	-.025 (-.037, -.013)	.0061	**<0.001**
T_EM_	-.054 (-.098, -.010)	.0224	**<0.016**
T_RV_	.171 (.117,.224)	.0273	**<0.001**
Age			
T_Na_	-.005 (-.007, -.002)	.0014	**.001**
T_CM_	.000 (-.001,.001)	.0004	.722
T_EM_	.001 (-.002,.004)	.0015	.373
T_RV_	.003 (.000,.007)	.0018	.089
Sex (female)			
T_Na_	.069 (.028,.111)	.0212	**.001**
T_CM_	.011 (-.001,.023)	.0061	.069
T_EM_	-.025 (-.069,.019)	.0148	0.267
T_RV_	-.055 (-.109, -.002)	.0727	**.042**

P-values in boldface are below the 0.05 threshold.

The effect of CMV on the estimated size of CD8 *T*_CM_, *T*_EM_, and *T*_Rv_ memory subsets was statistically significant. The strongest effect was seen on the *T*_Rv_ subset, with positive CMV serology accounting for an approximately 17% increase (*p*< 0.001), whereas the effects on the other subsets were much smaller (**Table 4**).

### CMV-specific T-cells dominate the late memory compartments in some but not most people

**Figure 8:**
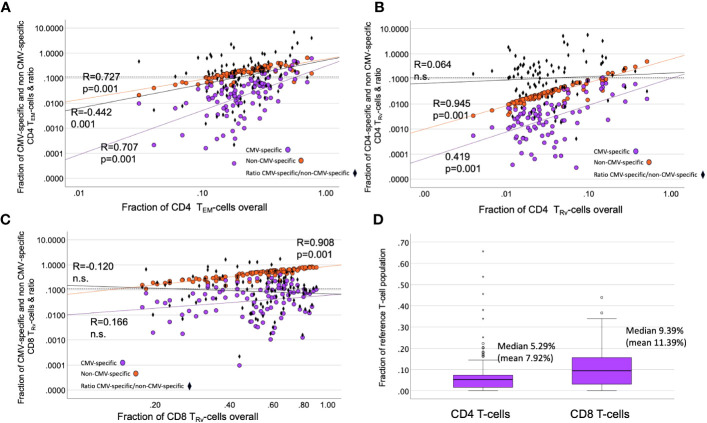
Contribution of CMV-specific T-cells to selected memory subsets. **(A–C)** The scatter plots show non-CMV-specific (orange) and CMV-specific T-cells (violet) *versus* the relative overall size (% of all CD4 or all CD8 T-cells) of the indicated memory compartments (T_Rv_ or T_EM_). The ratio of CMV-specific/non-CMV-specific T-cells is indicated as black diamonds. Black diamonds above the dotted horizontal line indicate proportions of CMV-specific T-cells >10% in the respective compartment. **(D)** The estimated proportion of CMV-specific CD8 T-cells (among all CD8 T-cells) is bigger than that of the CD4 T-cell response (among all CD4 T-cells).

While CMV-specific T-cell responses may be very large in some individuals (7), plotting the estimated percentages of CMV-specific and non-CMV-specific T-cells against the size of the CD4 *T*_EM_ and CD8 *T*_Rv_ compartments revealed that, even after extrapolating the CMV-specific T-cells, their contribution to overall compartment size was extremely variable, and, in particular, in CD8 T-cells it was quite small, illustrating the on average minor direct contribution of these cells to the overall memory subset size (**Figures 8A, B**). The estimated overall response size was similar to the responses recorded in the Sylwester 2005 study (dataset 2) among CD4 and CD8 T-cells (**Figure 8C**).

### The distribution of CMV-specific T-cell memory subsets appeared to be unrelated to the distribution of non-CMV-specific T-cells

We previously introduced a differentiation score (DSc) to capture the distribution of T-cells into different memory compartments in a single number (11). It is calculated by weighting the percentages of T-cells in the *T*_CM_, *T*_EM_, and *T*_Rv_ compartments where *T*_CM_ is multiplied by 1/3, *T*_EM_ by 2/3, and *T*_Rv_ by 1. The score is the sum of the weighted proportions, i.e.:


$$\text{DSc}=\sum_{i=0}^{3}{\text{\%Subset}}_{\text{i}}*\frac{i}{3}$$

We previously showed that larger CMV-specific responses tend to have higher differentiation scores (11). For the current analysis, we explored the correlations between the DSc of all CD4 T-cells, CMV-specific CD4 T-cells, and non-CMV-specific T-cells. There were strong correlations between all CD4 T-cells and non-CMV-specific CD4 T-cells (*R* = 0.956, *p* = 0.000) as well as all CD8 T-cells and non-CMV-specific CD8 T-cells (*R* = 0.980, *p* = 0.000). The correlations between CMV-specific and all or non-CMV-specific T-cells were at best weak to moderate (**Table 5**), indicating that it is not the degree of differentiation of CMV-specific T-cells (or functional consequences resulting from it) that drives the differentiation of non-CMV-specific T-cells. There was also only a weak to moderate correlation between the DSc of CMV-specific CD4 and CD8 T-cells (*R* = 0.424, *p* = 0.000). We further tested if the size of the CMV-specific CD4 or CD8 T-cell response had any effect on the DSc of all CMV-specific or non-CMV-specific T-cells and found a moderate to strong correlation between the size of the total CMV-specific CD4 response and the DSc of all CD4 T-cells (*R* = 0.580, *p*< 0.001). Such a correlation was not found in the CD8 compartment.

**Table 5:** Correlation of response size and differentiation score (DSc) between populations.

	Total CMV-specific CD4 response (%)	All CD4	CMV-specific CD4	Non-CMV-specific CD4	Total CMV-specific CD8 response (%)	All CD8	CMV-specific CD8	Non-CMV-specific CD8
CD4 total CMV-specific response	1	**.580**	**.264**	**.353**	-.036	.187	.052	.202
*p*-value		**<.001**	**.010**	**<.001**	.734	.071	.619	.051
DSc all CD4	**.580**	1	**.273**	**.956**	.088	**.393**	0.013	**.397**
*p*-value	**<.001**		**0.008**	**0.000**	.398	**0.000**	0.902	**0.000**
DSc CMV-specific CD4	.264*	**.273**	1	0.166	-.085	0.128	**.424**	0.134
*p*-value	**.010**	**0.008**		0.110	.414	0.218	**0.000**	0.197
DSc non-CMV-specific CD4	**.353**	**.956**	0.166	1	.110	**.376**	-0.013	**.379**
*p*-value	**<.001**	**0.000**	0.110		.291	**0.000**	0.900	**0.000**
CD8 total CMV-specific response	-.036	.088	-.085	.110	1	.102	.238	-.054
*p*-value	.734	.398	.414	.291		.327	.021	.604
DSc all CD8	.187	**.393**	0.128	**.376**	.102	1	**.430**	**.980**
*p*-value	.071	**0.000**	0.218	0.000	.327		**0.000**	**0.000**
DSc CMV-specific CD8	.052	0.013	.424	-0.013	.238*	**.430**	1	**.343**
*p*-value	.619	0.902	**0.000**	0.900	**.021**	**0.000**		**0.001**
DSc non-CMV-specific CD8	.202	**.397**	0.134	**.379**	.054	**.980**	**.343**	1
*p*-value	.051	**0.000**	0.197	0.000	.604	**0.000**	**0.001**	

Bold values mark significant correlations (p<0.05).

## Discussion

For the present report, we explored the extent to which CMV infection drives memory T-cell differentiation within and outside of the CMV-specific T-cell compartment. A strong effect of CMV on peripheral blood lymphocyte subset distributions, including strong skewing of T-cell subsets towards a late(r) differentiation stage, has been reported by many authors (12, 13). Looking in more detail, however, in almost 100 CMV+ individuals, the present analysis showed that the memory subset distribution of the overall CD4 and CD8 T-cell compartment is strongly skewed only in a minority of CMV-infected people. Rather than a shift of the entire curve (indicating that all peripheral blood T-cells are affected in an unspecific way), we observed a larger spread of the size of CD4 *T*_EM_ and CD8 *T*_Rv_ subsets with much increased values in a minority of individuals.

To provide a more objective comparison between CMV- and CMV+ people and in analogy to our previous report on response sizes and outliers among CMV-specific T-cell responses (10), we determined non-parametric boundaries for outliers as upper quartile (UQ) + 1.5 * interquartile range (IQR) in CMV- people and then checked if the corresponding percentages of these subsets in CMV+ people were outliers or not. Based on these outlier limits, the percentage of the *T*_Rv_ subset was most likely to be an outlier with respect to both CD4 and CD8 T-cells. However, in almost two-thirds of CMV+ people, none of the subset percentages were outliers irrespective of T-cell compartment. While such skewed distributions may be less frequent in CMV- people, our data shows that the skewing can be just as strong in the absence of CMV infection. We acknowledge that there are some observable changes in CMV+ people, in particular among the CD8 T_Rv_ population (**Figure 3B**), that do not exceed the outlier threshold. These more subtle changes are not considered by our approach, which may be a limitation. However, it is still true that subset sizes up to the threshold are not uncommon among CMV- people.

To understand if the perceived differences between CMV- and CMV+ people with respect to the overall distribution of peripheral blood T-cells into the canonical *T*_Na_, *T*_CM_, *T*_EM_, and *T*_Rv_ compartments were the effect of (predominantly late differentiated) CMV-specific T-cells simply mixing with non-CMV-specific T-cells, we had to estimate both the size and the memory subset distribution of all CMV-specific T-cells in dataset 1. This included revisiting dataset 2 for testing the ability of the protein-specific responses recorded in dataset 1 to predict the entire CMV-specific T-cell response. As a result of optimizing the selection of proteins to predict the entire CMV-response as closely as possible, we were able to adopt the mechanism for estimating the overall response proportions as originally published in 2005 (7). We are aware that the individuals analyzed in the original study were a different mix of genetic backgrounds (HLA background); however, there was no alternative dataset to be used. We believe that this will only make a small difference since there are, to our knowledge, no reports showing that CMV-specific T-cell proportions significantly differ between populations of different biogeographical ancestry.

Following the estimation of the size and memory subset distribution of the entirety of CMV-specific CD4 and CD8 T-cells found in the peripheral blood of CMV+ individuals, we subtracted the CMV-specific portion of each memory compartment from its overall size (measured by a simple surface phenotype stain) in terms of CD4 or CD8 T-cell percentages, which left us with an estimate of the percentages of non-CMV-specific T-cells in each compartment. The distribution of these supposedly non-CMV-specific T-cells both among CD4 and CD8 T-cells still looked quite different from the memory subset distributions found in CMV- people. We also compared CD4 and CD8 T-cell memory subset distributions between individuals that did or did not have outlier responses with respect to the CD4 *T*_EM_ and *T*_Rv_ and the CD8 *T*_Rv_ compartments, respectively. This comparison showed that the distribution of non-CMV-specific memory subsets in those without outliers was more similar to the ones in CMV- people. To explore if the remaining differences were at least partly due to differences in age and sex between the CMV- and CMV+ groups, we used logistic regression. This revealed the effects of age and sex in addition to CMV serology on the extrapolated sizes of the non-CMV-specific *T*_CM_, *T*_EM_, and *T*_RV_ compartments. Importantly, it confirmed a significant positive effect of CMV serostatus on the CD4 *T*_EM_ and CD8 *T*_CM_, *T*_EM_, and *T*_RV_ compartments. The detected significant negative effect of female sex on the CD4 *T*_EM_ and CD8 *T*_Rv_ populations is in agreement with the fact that, in women, peripheral blood T-cells tend to show less terminal differentiation profile than in men (11).

The contribution of CMV-specific T-cells to the most differentiated compartments was visualized by plotting the estimated contributions of CMV-specific and non-CMV-specific CD4 *T*_EM_, CD4 *T*_RV_, and CD8 *T*_EM_ cells to the respective overall memory compartments (**Figure 8**). This showed that, in some cases, CMV-specific T-cells only made a minimal contribution to a very large *T*_EM_ or *T*_Rv_ compartment. In some cases, large CMV-specific responses went with a small proportion of non-CMV-specific T-cells and *vice versa*, indicating that the effect on the non-CMV-specific T-cells is not driven by the size of the CMV-specific response.

Because more CMV protein coding content has been discovered since the original analysis (14), the method for estimating overall CMV-specific response size devised in the original analysis in 2005 may result in an underestimation of overall response size. The use of a single cytokine readout, i.e., IFN-γ, rather than a combination of activation markers is also likely to underestimate a response size. This is because not all CMV-specific T-cells will produce IFN-γ in response to stimulation with CMV antigens or other antigens (15). Studies using MHC tetramers for single CD8 T-cell epitopes suggested that only around approximately 60% of T-cells, on average, will produce IFN-γ (16, 17). This indicates that we may still have underestimated the proportion of CMV-specific T-cells in our sample. With respect to the memory subset distribution of the entire CMV-specific T-cell response, we chose to use the weighted average for each subset across all positive responses to estimate the overall CMV-specific memory subset distribution in each individual. This approach accounts for the fact that larger responses make a larger contribution to the overall distribution. If the CMV-specific T-cell response was still much larger than our estimate, the “corrected” memory subset distribution of non-CMV-specific T-cells would be different; however, the apparent effect of CMV on non-CMV-specific T-cells would not disappear.

The wide variation of overall memory compartment distributions across CMV+ individuals as well as the lack of correlation between the relative sizes (in % of CD4 or CD8 T-cells) of corresponding memory subsets among CMV-specific and non-CMV-specific T-cells suggested that different mechanisms drive the effect of CMV infection on these populations. In order to be able to compare overall T-cell differentiation between populations more comprehensively, we performed a bivariate correlation analysis using an overall CMV-specific response size and the previously introduced T-cell differentiation index (DSc) (11) for the various populations of interest. These analyses confirmed that the differentiation of CMV-specific T-cells had no noticeable effect on the differentiation of non-CMV-specific T-cells. The finding that the overall proportion of CMV-specific CD4 T-cells correlated moderately with the differentiation score of all CD4 T-cells (**Table 5**) is in agreement with our previous report of a moderate positive and a moderate negative correlation between the size of the CMV-specific CD4 T-cell response and the size of the *T*_CM_ and *T*_Rv_ compartments (referred to as *T*_EMRA_ in the cited publication), respectively, of the tuberculin-specific CD4 T-cell response (18). However, these weak to moderate correlations offer no explanation as to what the mechanism behind these correlations might be and if these are direct or indirect effects. We cannot exclude that the situation might look different if we had access to other compartments—for example, secondary lymphoid organs or the lung, where many CMV-specific T-cells are found as shown in rhesus macaques (19). This is a limitation that most studies into human pathogen-specific T-cells face.

Our analysis was designed to explore if the skewed overall memory T-cell subset distribution observed in CMV+ individuals is simply the result of mixing a population of CMV-specific T-cells with large *T*_EM_ and *T*_EMRA_ components with a population of non-CMV-specific T-cells, many of which are *T*_Na_ or *T*_CM_. While we may still have somewhat underestimated the size and skewness of the memory subset distribution of CMV-specific T-cells, we conclude that the pronounced skewing of the overall memory subset distribution observed in approximately 30% of CMV+ people cannot simply be explained by the presence of CMV-specific T-cells in the mix. The fact that strong overall memory subset skewing is only present in a minority of CMV+ people suggests that there must be predisposing factors rendering individuals susceptible to these changes if they are infected with CMV. While the mechanisms proposed by Fletcher et al. (4) could, in theory, be effective in this minority of individuals, it remains unclear why the size of the effect would differ so much among CMV+ people. Moreover, Fletcher’s explanation would appear to imply that (reactivated) CMV is ordinarily (or at least frequently) present in lymph nodes at the time when T-cells for other infectious antigens are primed. While it is generally accepted that CMV infection causes immune activation driving immune system differentiation, a convincing correlate of a general CMV-driven immune activation beyond the lymphocyte compartment has remained elusive. In the future, it will be important to focus on those individuals in whom CMV has the largest impact on shaping the T-cell compartment as these changes are associated with immune system aging.

## Data availability statement

The raw data supporting the conclusions of this article will be made available by the authors, without undue reservation.

## Ethics statement

The studies involving humans were approved by UK National Research Ethics Service (NRES) ‘London Centre’ (Reference 13/LO/1270). The studies were conducted in accordance with the local legislation and institutional requirements. The participants provided their written informed consent to participate in this study.

## Author contributions

SF: Conceptualization, Methodology, Visualization, Writing – original draft, Writing – review & editing. BR: Data curation, Formal Analysis, Methodology, Software, Writing – original draft, Writing – review & editing. OF: Supervision, Writing – review & editing. AP: Formal Analysis, Methodology, Writing – review & editing. LP: Supervision, Writing – review & editing. FK: Conceptualization, Formal Analysis, Investigation, Methodology, Project administration, Supervision, Visualization, Writing – original draft, Writing – review & editing.

## Supplementary material

Supplementary Table 1 Explanation of weighted *versus* simple averages for memory subset distributions.

Supplementary Figure 1 Representative flow plots illustrating the gating strategy.

Supplementary Figure 2 Relative T-cell memory compartment distributions among all CD4 T-cells, CMV-specific CD4 T-cells, and non-CMV-specific CD4 T-cells.
