# Supplementary material for: Marked skewing of entire T-cell memory compartment occurs only in a minority of CMV-infected individuals and is unrelated to the degree of memory subset skewing among CMV-specific T-cells
Source: Front Immunol. 2023 Oct 26;14:1258339. doi: 10.3389/fimmu.2023.1258339 (PMC10639168; doi:10.3389/fimmu.2023.1258339)
Supplement: Supplementary Table 1 — Explanation of weighted versus simple averages for memory subset distributions. [file Table_1.docx]

**Table S1: Explanation of *Weighted* versus *Simple Averages* for memory subset distributions**

| **T-cell subset** | **CMV protein Tube** | **Na (%)** | **CM (%)** | **EM (%)** | **Rv (%)** |  |
| --- | --- | --- | --- | --- | --- | --- |
| CD4 | 1 (protein 1) | … | … | … | … |  |
| CD4 | 2 (protein 2) | … | … | … | … |  |
| CD4 | 3 (protein 3) | … | … | … | … |  |
| CD4 | … | … | … | … | … |  |
| CD4 | 12 (protein 12) | … | … | … | … |  |
| CD4 | 13 (protein 13 and 14) | … | … | … | … |  |
| CD4 | 14 (protein 15 and 16) | … | … | … | … |  |
| (1) | **Column totals** | **Na_sum_** | **CM_sum_** | **EM_sum_** | **Rv_sum_** | Row total: **Mem_sum_** = (Na_sum_ + CM_sum_ + EM_sum_ + Rv_sum_) = Overall CMV-specific response |
|  | Weighted averages (division of columns totals by overall response size) | Na_sum_/ Mem_sum_ | CM_sum_/Mem_sum_ | EM_sum_/ Mem_sum_ | Rv_sum_/ Mem_sum_ |  |
| (2) | **Column averages** (=simple averages) | **Na_avg_** | **CM_avg_** | **EM_avg_** | **Rv_avg_** |  |
