# Supplementary figures and images for: Marked skewing of entire T-cell memory compartment occurs only in a minority of CMV-infected individuals and is unrelated to the degree of memory subset skewing among CMV-specific T-cells
Source: Front Immunol. 2023 Oct 26;14:1258339. doi: 10.3389/fimmu.2023.1258339 (PMC10639168; doi:10.3389/fimmu.2023.1258339)

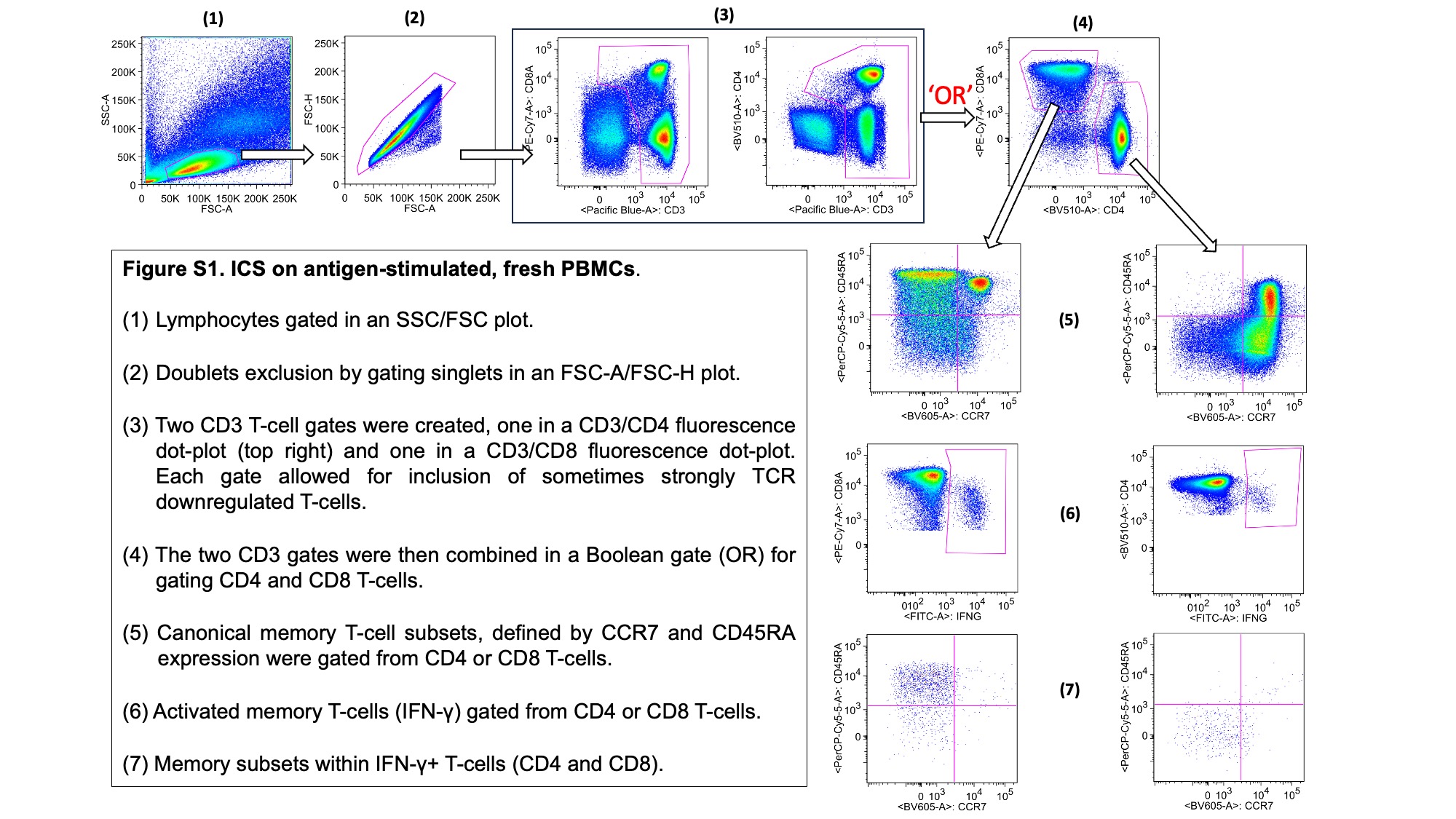

Supplement: Supplementary Figure 1 — Representative flow plots illustrating the gating strategy. [file Image_1.jpeg]

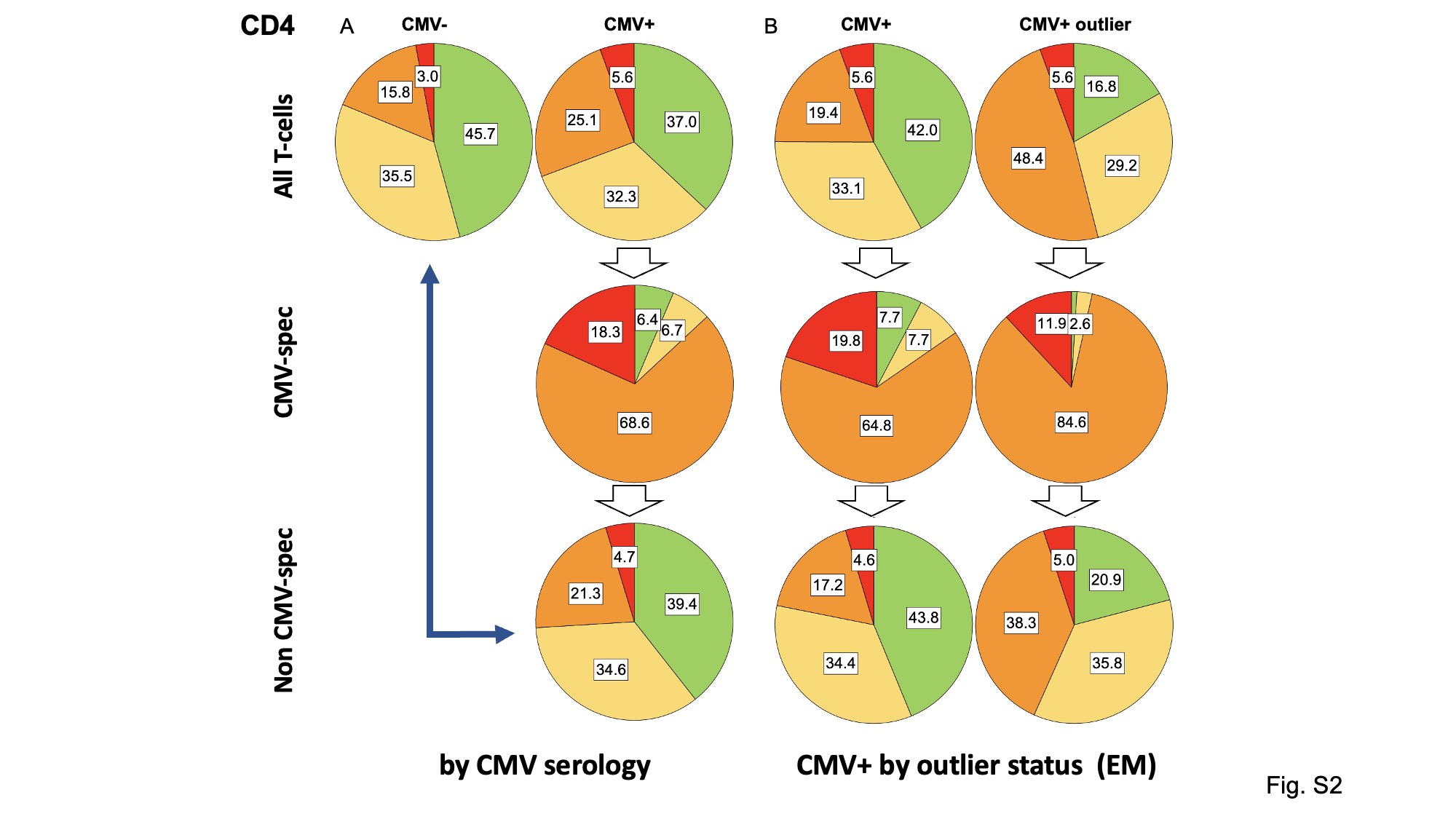

Supplement: Supplementary Figure 2 — Relative T-cell memory compartment distributions among all CD4 T-cells, CMV-specific CD4 T-cells, and non-CMV-specific CD4 T-cells. [file Image_2.jpeg]
